# An Interval Prediction of Chloroprene Rubber Crack Propagation Characteristics Based on Thermal Accelerated Aging

**DOI:** 10.3390/polym15112445

**Published:** 2023-05-25

**Authors:** Shengwen Yin, Yu Bai, Feng Kong, Zhonggang Wang, Congcong Fang

**Affiliations:** Key Laboratory of Traffic Safety on Track, Ministry of Education, School of Traffic & Transportation Engineering, Central South University, Changsha 410082, China; shengwen@csu.edu.cn (S.Y.); baiyu888@csu.edu.cn (Y.B.); fengkong@126.com (F.K.); wangzg@csu.edu.cn (Z.W.)

**Keywords:** thermal aging, fatigue life, Arrhenius equation, uncertainty, rubber

## Abstract

Thermo-oxidative aging plays an important role in changing the properties of rubber materials; it significantly decreases the fatigue life of air spring bags and further causes safety hazards. However, due to the great uncertainty of rubber material properties, an effective interval prediction model has not been established considering the effect of aging on airbag rubber properties. To solve the problem, this study proposes an interval parameter correlation model that can more accurately describe rubber crack propagation characteristics by considering material uncertainty. Furthermore, an aging prediction model of the rubber crack propagation characteristic region is established based on the Arrhenius equation. The effectiveness and accuracy of the method are verified by comparing the test and prediction results under the temperature spectrum. The method can be used to determine the variations in the interval change of the fatigue crack propagation parameters during rubber aging and can guide fatigue reliability analyses of air spring bags.

## 1. Introduction

Owing to their superior vibration isolation performance, noise reduction, and cushioning performance, rubber materials are used in a wide range of industrial components, such as air springs, automobile tires, seals, and shock pads [1,2]. However, the fatigue properties of rubber parts tend to deteriorate during long-term service, which has an impact on their lifetime and reliability [3]. Today, the methods for studying the fatigue life of rubber parts mainly include crack initiation and crack propagation methods. These methods have advantages and disadvantages in different engineering applications [4]. The crack initiation method is mainly used to perform fatigue analyses on rubber parts under heavy loads. The fatigue life of some rubber parts is nearly equal to the time of crack initiation [5]. However, for air spring bags, chloroprene rubber (CR) material plays the role of sealing and protecting the airbag cord, rather than the main load-bearing structure. Only when a crack in the rubber matrix is extended to a certain limit will the reliability and safety of the components be affected [6]. 

Based on fracture mechanics, several researchers have reported a series of basic mechanistic studies on the crack propagation properties of rubber materials. These studies have provided a theoretical foundation for predicting the fatigue life of rubber parts [7,8,9]. On this basis, researchers have evaluated the fatigue performance of rubber components. Peng et al. investigated the effects of the crack orientation angle and main tensile ratio on the crack energy density and predicted the fatigue life of rubber materials [10]. Wu et al. studied the fatigue durability characteristics of aircraft tires and predicted the effects of the tread groove structure and slip angle on the tire fatigue wear characteristics by combining tire performance tests and finite element analysis methods [11]. However, these studies ignored the effect of the aging rubber materials on the fatigue life of the rubber parts. The actual service fatigue life of air spring airbags is much lower than the life tested by the fatigue-testing machine. This is mainly due to the change in the molecular structure after the aging of the airbag rubber material, which leads to a decline in the ability of the rubber molecules to resist fatigue load. In engineering, rubber materials are typically susceptible to service conditions, which results in the serious deterioration of their rubber properties, such as thermal-oxidative aging, radiation exposure, and chemical corrosion. Thermal aging has a considerable effect on many properties of rubber, such as the elongation of their fatigue characteristics [12], hardness [13], stiffness [14], and rupture [15]. Therefore, to ensure the safety of rubber parts in long-term service, the effect of rubber aging on these fatigue properties needs to be studied to avoid potential safety hazards.

The natural aging test is one of the effective methods for evaluating the performance of aging rubber materials; however, this method is time-consuming. Therefore, the thermal accelerated aging test is widely chosen to rapidly study the performance of aging rubber materials [16,17,18]. Currently, many studies are being conducted on the fatigue properties of rubber parts considering aging factors. Olejnik et al. [19] and Kashi et al. [20] explored the influence of aging on the fatigue characteristics of various rubber materials through high-temperature accelerated aging, and their research results showed that the fatigue crack growth rate of rubber after aging would seriously deteriorate. However, they only analyzed the impact of aging and did not further predict or model the degradation trend of the aging rubber crack propagation rate. Moon et al. [21,22] predicted the durability life of tires under actual driving conditions by exploring the influence of aging on strain energy density. A prediction model for rubber crack propagation parameters that is suitable for the Arrhenius equation has not yet been established. Furthermore, the mechanical properties of rubber products are inevitably uncertain, owing to manufactory errors and other factors. The methods for uncertainty analyses are typically probabilistic and fuzzy, as are interval methods [23,24]. The uncertainties identified using the probability method were more reliable. However, sufficient statistical information is typically lacking, thereby making it difficult to obtain the probability distribution function of the parameters. Conversely, a non-probabilistic interval analysis method is more appropriate for cases with limited information about uncertain parameters. Thus, to reduce the workload of the existing uncertainty prediction methods and simplify the prediction process, this study introduces an interval analysis method to characterize the uncertainty of rubber crack propagation performance.

Based on the literature survey, though lots of studies have been conducted on the life prediction of rubber products, some important issues still remain unsolved: (a) the method for predicting the crack propagation of rubber products considering both uncertainties and material deterioration is unreported; and (b) in traditional interval analysis methods, the parameters for modelling the mechanical properties of rubber are assumed to be independent. However, one of the major shortcomings based on independent assumptions is the relatively large overestimation caused by the so-called ‘‘wrapping effect’’ [25]. Therefore, it is desirable to develop a life prediction method that can effectively estimate the crack propagation of rubber materials under interval uncertainties. 

The aim of this study was to develop an effective method for predicting the fatigue crack propagation parameter deterioration during the aging process of air spring airbag rubber materials. To this end, the degradation of the fatigue crack propagation behavior and the dispersion of the property distribution of aged CR at different accelerated temperatures were statistically analyzed. A correlation model of the rubber crack propagation parameter interval was proposed to improve the interval propagation problem caused by the independent interval parameters. Then, combined with the Arrhenius equation, a prediction model of the crack propagation rate interval of aging rubber material was established. The validity of the accelerated aging test was ensured based on the principle of constant activation energy. Finally, the accuracy of the life prediction method was demonstrated by comparing it to the results of the fatigue crack propagation testing.

## 2. Materials and Experimental Methods

### 2.1. Material

In this study, rectangular chloroprene rubber plates (thickness = 2 mm) were used as the test material, which were obtained by cutting and layering the commercial air spring airbag of a high-speed train. The main mechanical properties and filling composition of the material are shown in Table 1 and Table 2, respectively. A circulating air oven was used to accelerate the aging of the plates at four different temperatures during specific aging times to obtain different levels of aging of the rubber material. At the laboratory temperature (296 K ± 2K), the tensile test and fatigue crack propagation test were conducted on the aged rubber plates, according to relevant test standards, to obtain the change rule of the fatigue performance of the aging rubber.

### 2.2. Accelerated Aging Tests

Accelerated aging tests were performed according to the ISO 188:2011 standard [26]. An air-circulating oven (QLH-100) with an active ventilation function was selected to ensure a constant level of oxygen, thereby providing constant aging conditions. In order to ensure the efficiency of the accelerated test and avoid the influence of an excessively accelerated aging temperature on the aging mechanism of the rubber, the accelerated temperature range was selected to be from 343 K to 373 K. In the range of this accelerating temperature, rubber materials usually show an aging rate that increases by 0.5 times to 1 times for every 10 K rise in temperature [27,28]. This is because the air spring airbag usually appears with obvious cracks and continues to expand after one and a half years of service. In order to predict the crack propagation performance of air spring airbag rubber material after one and a half years of service, the maximum aging time at each accelerated aging temperature was determined according to this principle. The specific thermal aging conditions are listed in Table 3. All the aging temperature deviations were controlled within ±1.2 K. When the set aging time was reached, the rubber samples were taken out of the oven and then cooled on a flat surface. The aging rubber plates were maintained at room temperature for at least 24 h and their physical properties were tested.

### 2.3. Tensile Tests

Tensile tests were carried out to obtain stress–strain curves of the aged rubber materials, which were used to calculate the tearing energy of the crack tip of the aged rubber. According to ASTM D412-a [29], the aged rubber plates were cut into dumbbell-shaped rubber specimens, and the static tensile tests were carried out on the cut specimens. Eliminating the Mullin effect on rubber materials with repeated tensile tests is necessary to obtain accurate tensile property data on the rubber materials. Before the actual tensile test, cyclic tensile testing (rate: 50 mm/min) was conducted within the range of the strain to be measured until the tensile test curve was stable under repeated loading. Note that the maximum strain of the air spring bag rubber was typically less than 0.4; therefore, the test strain range was set from 0 to 0.6. This method gradually stabilized the molecular chain structure of the rubber, thereby eliminating the Mullin effect on the rubber materials [30].

### 2.4. Fatigue Crack Propagation Test

**Figure 1:**
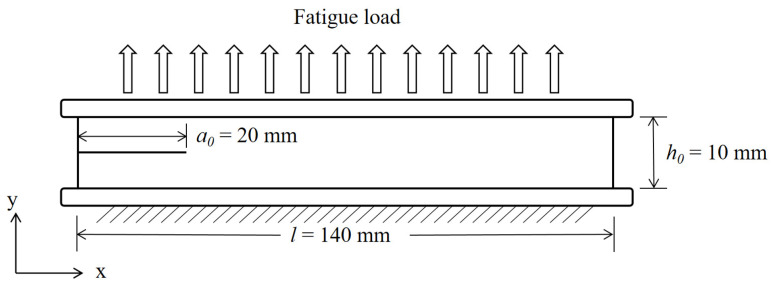
Dimensions of the pure shear rubber specimen (all dimensions in mm).

Pure shear rubber specimens have the advantages of a constant crack propagation rate under the condition of constant amplitude fatigue-loading cycles [31]. Therefore, pure shear rubber specimens were used in this study to investigate the crack propagation properties of aging rubber. Figure 1 shows the theoretical structural dimensions of the specimen. To ensure the validity of the experimental results, the test area of the sample was in the pure shear state. The dimensions of the specimen were required to meet the appropriate length to height ratio ($a_{0}\geq 2h_{0}$) [32]. The prefabricated crack length ($a_{0}$) and original height ($h_{0}$) of the pure shear specimen were set as 20 mm and 10 mm, respectively.

The variation in the specimen crack length within the effective test area is independent of the crack-tearing energy. The tear energy can be calculated from the strain energy density (ω) at the far end of the crack and the original height ($h_{0}$) of the specimen, satisfying Equation (1) [31]:

(1) $$G=h_{0}\cdot \omega$$

According to the stress–strain results from the tensile tests on the rubber specimens, the corresponding strain energy density can be obtained via integral calculation, as shown in Equation (2).

(2) $$\omega =\int_{0}^{\varepsilon }\sigma d\varepsilon$$

**Figure 2:**
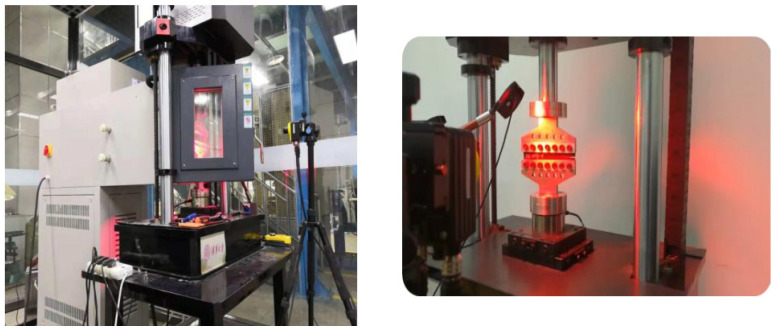
Fatigue testing equipment.

**Figure 3:**
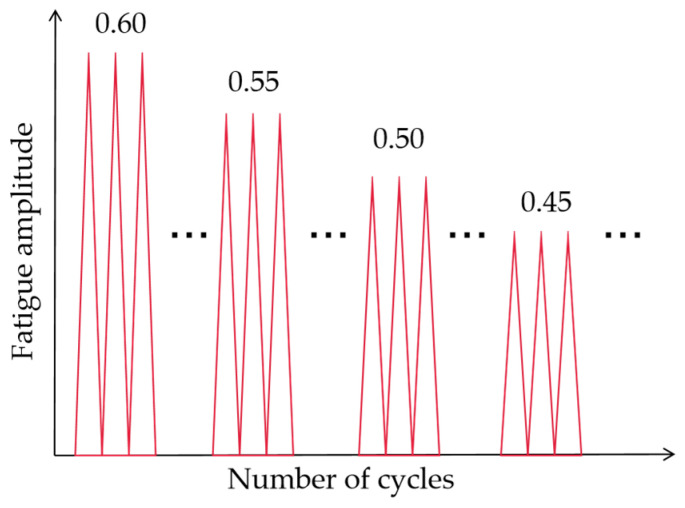
Diagram of fatigue cycle load.

According to the ASTM-D813-07 [33], the uniaxial cyclic tensile tests on the pure shear rubber samples were performed using servo fatigue-testing equipment named MTS Landmark (Figure 2). Previous studies have shown that, when the temperature is constant and the fatigue cycle frequency is higher than 1 Hz, the change in the cycle load frequency does not affect the fatigue properties of rubber materials [34]. However, the high frequency of the fatigue load will lead to a temperature increase in the rubber specimen and affect the experimental results. In order to obtain the data on the crack length and ensure the temperature consistency of the fatigue test, the test was carried out in a semi-closed incubator. Through the pre-test, it was found that, when the fatigue frequency was less than or equal to 4 Hz, the surface temperature rise in the sample was less than 2 K. Therefore, the fatigue displacement load frequency of the test was set at 4 Hz and the stress ratio was R = 0. The fatigue load shown in Figure 3 was applied to the rubber specimen. The crack area of the specimen was photographed with an ultra-HD camera to identify the change in the crack length. Thus, the crack propagation rate of the rubber material under fatigue loads of various amplitudes can be obtained.

## 3. Prediction Model and Theory of Rubber Aging Interval Parameters

This section establishes a model for predicting the crack propagation regions of aging rubber materials. Because the traditional interval analysis method for material parameters does not consider the correlation of the parameters, it inevitably leads to the phenomenon of overestimation. Therefore, to avoid this problem, a correlation model for the crack propagation interval parameters of the rubber materials is established. The description accuracy of the rubber fatigue characteristic area is improved. In addition, using the aging prediction method based on the Arrhenius Equation, an aging prediction model for the rubber crack propagation characteristic region is established. It is then used to predict the deterioration trend of the fatigue crack propagation properties interval of the aging rubber materials.

### 3.1. Interval Correlation Model of Rubber Crack Propagation Parameters

To construct the correlation model of the rubber crack propagation interval parameters, the phenomenon of overestimation caused by the independent interval parameters calculated by the traditional Monte Carlo method is demonstrated. Then, the internal causes of the problem are analyzed to determine the constraint relationship between the interval parameters of the linear equations. Finally, the basic theory of rubber crack propagation is introduced based on the constraint relationship between the obtained interval parameters of the linear equations, and a rubber crack propagation interval parameter association model is established.

#### 3.1.1. Interval Parameter Identification Method Based on the Monte Carlo Method

Lake et al. studied the trend between the tearing energy (G) and fatigue crack propagation rate (da/dN) of rubber materials, and observed that, in the tearing energy range that can lead to stable crack propagation, Equation (3) is satisfied between G and da/dN [9]. According to Equation (3), a linear relationship exists between G and da/dN in the logarithmic coordinate system. Therefore, the interval analysis of the rubber crack propagation parameters can be transformed into an interval parameter analysis of the linear equation.

(3) $$\frac{da}{dN}=a\cdot G^{b},\ln(\frac{da}{dN})=\ln a+b\cdot \ln G$$

In Equation (3), a and b are the performance parameters that reflect the crack propagation characteristics of the rubber materials.

**Figure 4:**
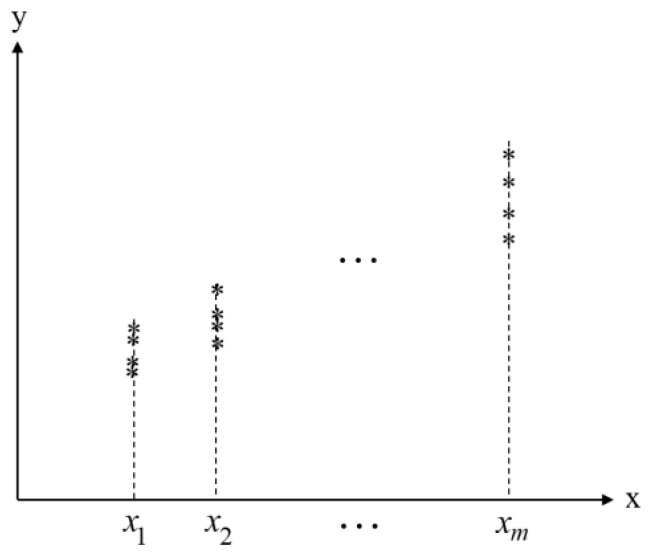
Schematic of restricted area.

The Monte Carlo method can be used to solve the range of the parameter variation in the linear equation in the linear region. Taking the linear region shown in Figure 4 as an example, the interval expansion phenomenon caused by the independent interval parameters of the linear equation and its causes are analyzed.

**Figure 5:**
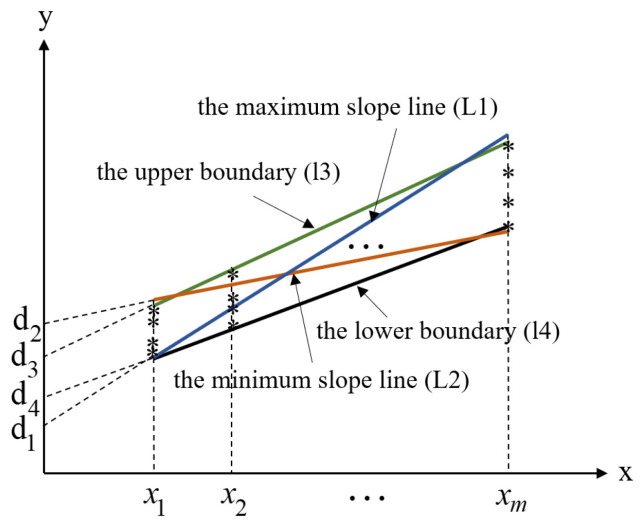
Fitting results of each characteristic line of regional data.

In Figure 4, $x_{i}(i=1,2,3,\ldots ,m)$ is in the range of [$x_{1}$,$x_{m}$]. The Monte Carlo method is used to randomly select $y(x_{i})$ values in the illustrated area and the random values are linearly fitted. Monte Carlo random sampling is carried out in a cycle until the limit values of parameters c and d of the linear equation stabilize. The value ranges of parameters c and d are obtained as $c\in [c_{1},c_{2}]$ and $d\in \left[d_{2},{\;d}_{1}\right]$. The limits for parameters c and d are obtained when the maximum slope line ($L_{1}$) and minimum slope line ($L_{2}$) are as shown in the region of Figure 5, respectively. Additionally, the equations for $L_{1}$ and $L_{2}$ are expressed as follows:

(4) $$L_{1}:y_{1}=c_{1}\cdot x+d_{1}$$

(5) $$L_{2}:y_{2}=c_{2}\cdot x+d_{2}$$

According to Equations (4) and (5), the limits of the linear region at $x_{1}$ and $x_{m}$ can be obtained, respectively. Thus, the upper and lower boundary equations of the linear region are obtained, respectively, as follows:

(6) $$L_{3}:y_{3}=c_{3}\cdot x+d_{3}$$

(7) $$L_{4}:y_{4}=c_{4}\cdot x+d_{4}$$

where $L_{3}$ and $L_{4}$ are the upper and lower boundaries of the linear region, respectively. The parameters are defined as follows:

(8) $$\{\begin{array}{c} c_{3}=\frac{y_{1}(x_{m})-y_{2}(x_{1})}{x_{m}-x_{1}},d_{3}=\frac{x_{m}\cdot y_{1}(x_{1})-x_{1}\cdot y_{2}(x_{m})}{x_{m}-x_{1}}\\ c_{4}=\frac{y_{2}(x_{m})-y_{1}(x_{1})}{x_{m}-x_{1}},d_{4}=\frac{x_{m}\cdot y_{2}(x_{1})-x_{1}\cdot y_{1}(x_{m})}{x_{m}-x_{1}} \end{array}$$

**Figure 6:**
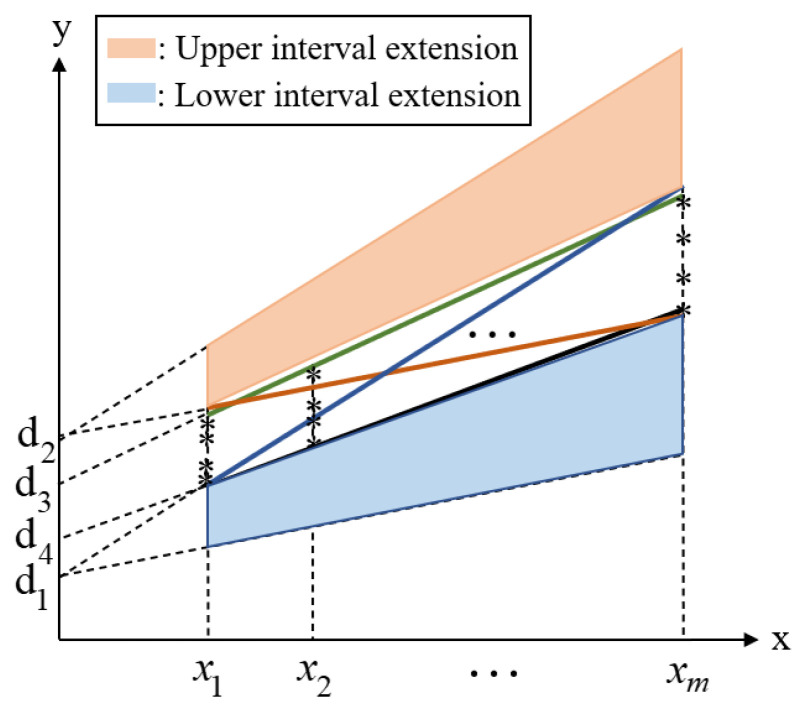
Overestimation caused by independent interval variables ***c*** and ***d***.

Notably, the values of the interval parameters ***c*** and ***d*** in the fitting region correspond to each other. For example, when parameter ***c*** assumes the maximum value $c_{2}$, parameter ***d*** can only assume the minimum value $d_{2}$. However, when the interval parameters ***c*** and ***d*** are independent variables, no mutual constraint limit is placed on the value range between the parameters. When the value of ***c*** is the maximum value $c_{2}$, if parameter ***d*** is arbitrarily valued in the range of $\left[d_{2},{\;d}_{1}\right]$, it will lead to an expansion of the upper interval, as shown in Figure 6. Similarly, when the value of ***c*** is the minimum value $c_{1}$, it leads to an expansion of the lower interval.

As shown in Figure 6, when the interval parameters ***c*** and ***d*** are regarded as independent interval variables, a clear overestimation is observed in the linear region described. Therefore, to avoid the phenomenon of interval propagation caused by ignoring the correlation between these interval variables, it is necessary to analyze the correlation of the linear equation parameters.

#### 3.1.2. Linear Interval Parameter Correlation Model

The constraint relationship between the interval parameters is established based on the analysis results of the phenomenon of overestimation due to the independent interval parameters, and a correlation model of the crack propagation interval parameters is constructed.

When the slope parameter ***c*** of the linear equation changes within the range of $\left[c_{1},c_{2}\right]$, the fitting line must be within the range of the fitted area to avoid overestimation. Because the fitting line is a straight line, the values of $y(x_{1})$ and $y(x_{m})$ of the fitted line only need to satisfy the following equations:

(9) $$\{\begin{array}{c} y(x_{1})\geq y_{1}(x_{1}),\\ y(x_{1})\leq y_{2}(x_{1}),\\ y(x_{m})\geq y_{2}(x_{m}),\\ y(x_{m})\leq y_{1}(x_{m}). \end{array}$$

In Equation (9), $y_{1}$ represents Equation (4), $y_{2}$ represents Equation (5), $y_{3}$ represents Equation (6), $y_{4}$ represents Equation (7), and $x_{1}$ and $x_{m}$ are the boundary values of the independent variable *x* in the fitted region, respectively. That is, the value of parameter *d* needs to satisfy the following:

(10) $$\{\begin{array}{c} d\geq y_{1}(x_{1})-c\cdot x_{1},\\ d\geq y_{2}(x_{m})-c\cdot x_{m},\\ d\leq y_{2}(x_{1})-c\cdot x_{1},\\ d\leq y_{1}(x_{m})-c\cdot x_{m}. \end{array}$$

By analyzing the boundary area shown in Figure 5, we can observe that, when $c\in [c_{1},c_{2}]$, the range of ***d*** needs to meet certain conditions to ensure that the fitting line is within the range of the region. These conditions can be divided into three cases:

I: For ***c***$\in [c_{3},c_{1}]$, because the value of parameter ***c*** is greater than the slope parameter $c_{3}$ of the upper boundary, the value of the fitting line is greater than that of the boundary line. Thus, the value of parameter ***d*** of the fitting line needs to meet the value of the fitting line at $x_{1}$, which is greater than the minimum value of the area, and the value of the fitting line at $x_{m}$, which is less than the maximum value of the area, that is:

(11) $$\{\begin{array}{c} d\geq y_{1}(x_{1})-c\cdot x_{1},\\ d\leq y_{1}(x_{m})-c\cdot x_{m}. \end{array}$$

II: For ***c***$\in [c_{4},c_{3}]$, because the value of parameter ***c*** is between the slopes of the upper and lower boundaries, the value of the fitting line is greater than that of the lower boundary, but less than that of the upper boundary. Therefore, the value of parameter ***d*** needs to meet the value of the fitting line at $x_{1}$, which is greater than the minimum value of the region, but less than the maximum value of the region, that is:

(12) $$\{\begin{array}{c} d\geq y_{1}(x_{1})-c\cdot x_{1},\\ d\leq y_{2}(x_{1})-c\cdot x_{1}. \end{array}$$

III: for ***c***$\in [c_{2},c_{4}]$, because the value of parameter ***c*** is less than the lower boundary slope parameter $c_{4}$, the value of the fitting line is less than that of the boundary line. Therefore, the value of parameter ***d*** needs to meet the value of the fitting line at $x_{1}$, which is less than the maximum value of the area, and the value of the fitting line at $x_{m}$, which is greater than the minimum value of the area, that is:

(13) $$\{\begin{array}{c} d\geq y_{2}(x_{m})-c\cdot x_{m},\\ d\leq y_{2}(x_{1})-c\cdot x_{1}. \end{array}$$

Based on the analysis results of the three cases above, a correlation model of the linear equation parameters can be established as follows:

(14) $$\{\begin{array}{c} c\in [c_{3},c_{1}],d=[y_{1}(x_{1}),-c\cdot x_{1},y_{1}(x_{m})-c\cdot x_{m}],\\ c\in [c_{4},c_{3}],d=[y_{1}(x_{1})-c\cdot x_{1},y_{2}(x_{1})-c\cdot x_{1}],\\ c\in [c_{2},c_{4}],d=[y_{2}(x_{m})-c\cdot x_{m},y_{2}(x_{1})-c\cdot x_{1}]. \end{array}$$

The interval correlation model of the rubber crack propagation parameters can be determined based on the established interval parameter correlation model of the linear equation. Considering the rubber crack propagation region as the fitting region, the maximum slope line ($L_{1}$) and the minimum slope line ($L_{2}$) of the crack propagation rate within the range are fitted by combining them with Equation (3), as shown in Equations (15) and (16), respectively:

(15) $$L_{1}:Y_{1}=\ln a_{1}+b_{1}\cdot \ln G$$

(16) $$L_{2}:Y_{2}=\ln a_{2}+b_{2}\cdot \ln G$$

In this case, Y is $\ln(\frac{da}{dN})$.

According to Equations (15) and (16), the upper and lower boundary equations of the crack propagation rate are obtained by fitting:

(17) $$L_{3}:Y_{3}=\ln a_{3}+b_{3}\cdot \ln G$$

(18) $$L_{4}^{}:Y_{4}=\ln a_{4}+b_{4}\cdot \ln G$$

where the parameters are defined as follows:

(19) $$\{\begin{array}{c} b_{3}=\frac{Y_{1}(\ln G_{M})-Y_{2}(\ln G_{1})}{\ln G_{M}-\ln G_{1}},\ln a_{3}=\frac{\ln G_{M}\cdot Y_{1}(\ln G_{1})-\ln G_{1}\cdot Y_{2}(\ln G_{M})}{\ln G_{M}-\ln G_{1}}\\ b_{4}=\frac{Y_{2}(\ln G_{M})-Y_{1}(\ln G_{1})}{\ln G_{M}-\ln G_{1}},\ln a_{4}=\frac{\ln G_{M}\cdot Y_{2}(\ln G_{1})-\ln G_{1}\cdot Y_{1}(\ln G_{M})}{\ln G_{M}-\ln G_{1}} \end{array}$$

The interval parameter correlation model of the rubber fatigue crack propagation can be obtained as follows:

(20) $$\{\begin{array}{c} b\in [b_{3},b_{1}],\begin{array}{c} \ln a\geq y_{1}(\ln G_{1})-b\cdot \ln G_{1}\\ \ln a\leq y_{1}(\ln G_{M})-b\cdot \ln G_{M} \end{array}\\ b\in [b_{4},b_{3}],\begin{array}{c} \ln a\geq y_{1}(\ln G_{1})-b\cdot \ln G_{1}\\ \ln a\leq y_{2}(\ln G_{1})-b\cdot \ln G_{1} \end{array}\\ b\in [b_{2},b_{4}],\begin{array}{c} \ln a\geq y_{2}(\ln G_{M})-b\cdot \ln G_{M}\\ \ln a\leq y_{2}(\ln G_{1})-b\cdot \ln G_{1} \end{array} \end{array}$$

Here, $G_{1}$ and $G_{M}$ represent the initial and final values of the tearing energy range of the fatigue test, respectively.

### 3.2. Interval Accelerated Aging Prediction Model

#### 3.2.1. General Arrhenius Equation

The basic theory behind rubber thermal acceleration tests is to accelerate the degradation process of the rubber materials using high-temperature thermal stress. The temperature change in a particular range only changes the aging rate, without changing the aging mechanism. Methods such as dynamic curves, function fitting, and neural networks are typically used to study the deterioration of rubber aging properties [35]. In this study, the efficiency of analyzing the effect of aging on the material properties of rubber is improved by using the dynamic curved straight method to simulate the aging performance, which is generally expressed as in Equation (21). Both sides of Equation (21) are converted to a logarithmic form simultaneously to obtain Equation (22):

(21) $$P=P_{0}\cdot e^{-K\cdot t^{\alpha }}$$

(22) $$\ln P=\ln(P_{0})-K\cdot t^{\alpha }$$

where P is the aging characteristic value of the rubber, $P_{0}$ is the initial value of P without aging, t represents the aging time, K is the reaction rate constant related to the thermally accelerated temperature, and α is the adjustment parameter of the linearity fitting. The linear correlation in Equation (22) is improved by adjusting the value of α (typically [0, 1]), which is determined by calculating the minimum value of the sum of the square errors of the test and predicted values.

The reaction rate constant K and thermal accelerated aging temperature T (Kelvin temperature) satisfy the Arrhenius equation, as shown in Equation (23) [36]:

(23) $$K=Z\cdot e^{-\frac{E_{a}}{RT}}$$

In Equation (23), Z is the regression constant, $E_{a}$ represents the activation energy (J/mol), and R represents a gas constant (8.314 J/mol).

A failure mechanism test was conducted using the K values to ensure the consistency of the accelerated aging mechanism. According to the relevant theory of the activation energy $E_{a}$ [31], when the failure mechanism is consistent, the $E_{a}$ values corresponding to each aging characteristic index are constants that do not change with temperature. The activation energy $E_{a}$ can be obtained using Equation (23). The activation energy $Ea_{T_{m}}$ at the *m*-th accelerated degradation temperature can be expressed as follows:

(24) $$Ea_{T_{m}}=\frac{\ln K_{T_{m+1}}-\ln K_{T_{m}}}{\left(\frac{1}{T_{m+1}}-\frac{1}{T_{m}}\right)}\cdot R$$

#### 3.2.2. Interval Crack Propagation Rate Prediction Based on Arrhenius Equation

According to the Arrhenius equation, each parameter in the interval parameter correlation model of the rubber crack propagation rate is considered as an aging characteristic index. The relationships between the parameters, accelerated aging temperature, and aging time are established. The prediction results of each aging characteristic index are inputted to the interval parameter correlation model of the rubber crack propagation, and a regional description model of the aging rubber crack propagation characteristics is constructed to predict the fatigue characteristics. The steps for establishing this interval aging prediction model are divided into the following:

(1) The fatigue crack propagation test is conducted on the rubber after accelerated aging, and the variation in the crack propagation rate of the accelerated aging rubber materials is obtained.
(2) According to the test results area of the rubber material with each degree of aging, the values of each parameter in the interval parameter correlation model of the rubber material crack propagation with this degree of aging are obtained through random sampling fitting, that is, the limits of each crack propagation parameter of the rubber materials: $\ln a_{1},\ln a_{2},b_{1},b_{2}$.
(3) The parameters ($\ln a_{1},\ln a_{2},b_{1},b_{2}$) are considered as the aging characteristic index, *P*. Because the parameters ($\ln a_{1},\ln a_{2}$) are negative, their absolute value is used as the aging characteristic index:
  (25) $$P^{1}=\left|\ln a_{1}\right|,P^{2}=\left|\ln a_{2}\right|,P^{3}=b_{1},P^{4}=b_{2}$$
(4) Using Equations (21)–(23), the relationship between each aging characteristic index ($P^{j},j=1,2,3,4$), aging temperature T, and aging time t is constructed, as follows:
  (26) $$P^{j}=P_{0}{}^{j}\cdot \exp(-K^{j}\cdot t^{\alpha^{j}}),K^{j}=Z^{j}\cdot e^{-\frac{E_{a}{}^{j}}{RT}}$$
(5) According to Equation (24), the consistency of the aging acceleration mechanism of each aging characteristic index $P^{j}$ is checked to ensure that the accelerated test temperature does not change the rubber aging mechanism. The validity of the aging prediction model is determined.
(6) The prediction equations for each parameter ($\ln a_{1},\ln a_{2},b_{1},b_{2}$) are introduced into the interval parameter correlation model of the rubber crack propagation. A prediction model for the crack propagation characteristic region of the aging rubber is established.

According to the above steps and the performance test results of the accelerated aging rubber, the interval prediction model of the aging rubber’s crack growth characteristics can be constructed. It is important to note that the prediction model is based on two assumptions: (1) The actual service temperature fluctuation of the rubber material should not be too large. Because the model is based on the test data at a room temperature of 296 K, theoretically, it can only predict the performance of the rubber material at this room temperature of 296 K after aging at ambient temperature for a period of time. (2) In order to simplify the characterization model of the rubber crack growth characteristics, a power function model is used to describe the relationship between the rubber crack growth rate and energy release rate, ignoring the tear threshold value of the rubber material. Therefore, this model is mainly applicable to the prediction of rubber crack propagation characteristics when the amplitude of the fatigue load is small and has a medium strain.

## 4. Results and Discussion

### 4.1. Results of the Fatigue Test

The test curve of the rubber crack propagation rate was obtained by controlling the strain amplitude using a servo fatigue-testing machine; however, da/dN, under the action of $G_{j}(j=1,2,\ldots ,M)$, could not be obtained directly from the test. First, using Equations (1) and (2), the strain energy and energy release rate G of the rubber specimen in the pure shear state at different strain amplitudes could be calculated. Then, the fatigue crack propagation test results of each rubber specimen were interpolated and fitted, using Equation (3) to determine the crack propagation rate $da/dN(G_{j})$.

Based on the rubber crack propagation test, when the tear energy of the non-aging rubber material was approximately 400 $J/m^{2}$, the crack propagation rate was close to a 1 × 10^−6^ mm/cycle. When the strain of the rubber with the deepest aging degree was 0.4, the maximum tear energy at the rubber crack tip was approximately 6500 $J/m^{2}$. Therefore, the test results for the crack propagation region with the energy release rate in the range of 400 $J/m^{2}$ to 6500 $J/m^{2}$ were selected as the modelling range.

**Figure 7:**
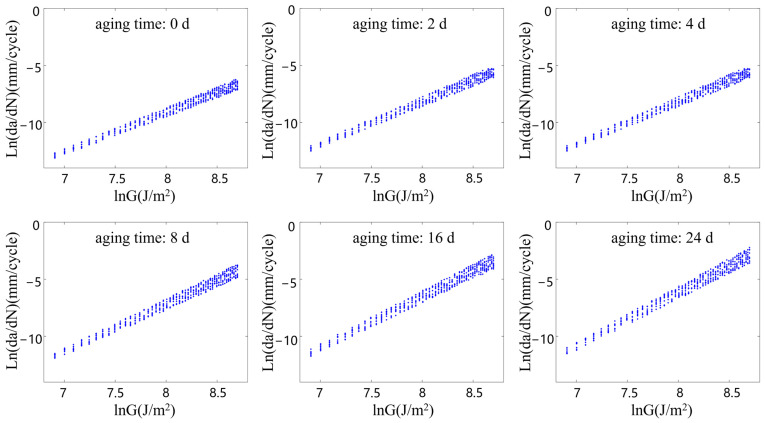
Rubber crack propagation performance after aging at 343 K.

**Figure 8:**
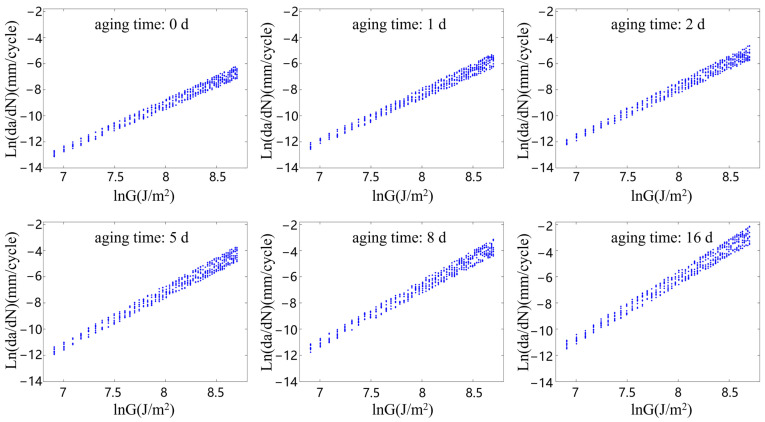
Rubber crack propagation performance after aging at 353 K.

**Figure 9:**
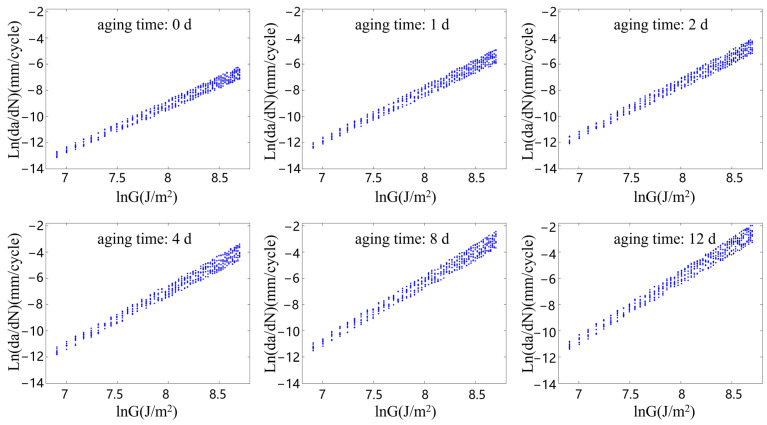
Rubber crack propagation performance after aging at 363 K.

**Figure 10:**
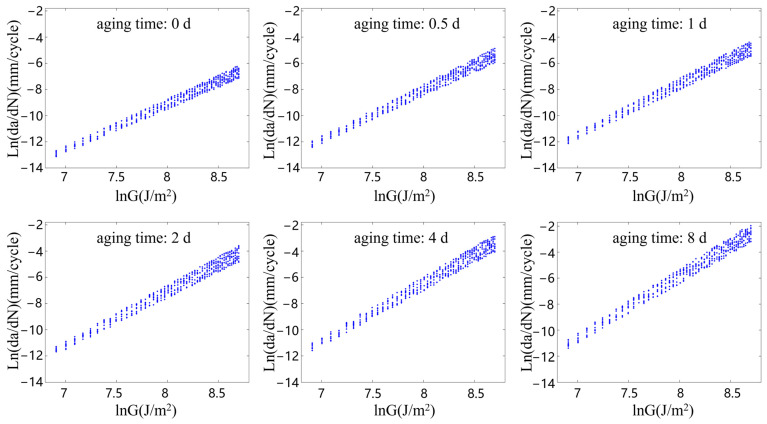
Rubber crack propagation performance after aging at 373 K.

Figure 7, Figure 8, Figure 9 and Figure 10 show the relationship between the ln(da/dN) and lnG of the rubber materials in logarithmic coordinates at different accelerated aging temperatures. The fatigue test results of the aging rubber all show an obvious linear trend between the crack propagation rate and energy release rate in the logarithmic coordinate system. Moreover, the crack propagation rate corresponding under the same G increased significantly with the degree of aging. The average crack propagation rate of the aging rubber at 373 K for eight days under the action of G = 3000 $J/m^{2}$ was a 0.0254 mm/cycle, which was 17.3 times higher than that of the unaged rubber. The curve slope of the crack propagation rate increased significantly after the aging of the rubber material. This phenomenon indicated that the rubber material became brittle and its fatigue resistance decreased. The aging rubber materials were more sensitive to changes in tear energy. Moreover, the discreteness of the rubber crack propagation rate varied with the degree of aging and the interval radius of the rubber crack propagation rate increased after aging. Clearly, thermal oxygen aging had a significant effect on the crack propagation properties of the rubber materials. The fatigue life of the rubber materials depended not only on the fatigue amplitude, but also on the degree of the aging of the material.

From the fatigue test data on the aging rubber shown in Figure 7, Figure 8, Figure 9 and Figure 10, the range of the limiting values of the crack propagation parameters of the rubber with different aging degrees was obtained via fitting, as shown in Table 4.

### 4.2. Interval Prediction of Crack Propagation

**Figure 11:**
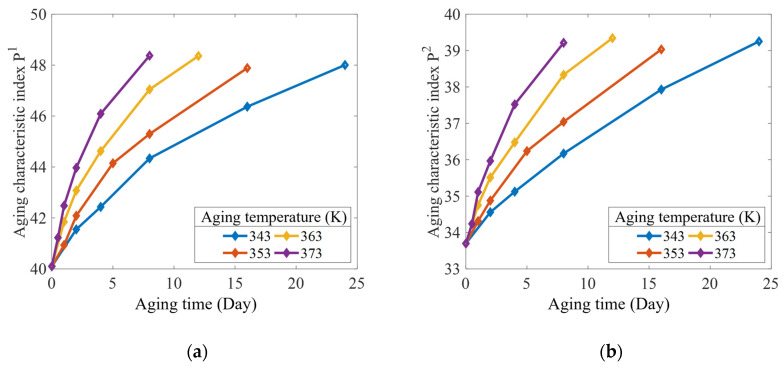
Accelerated aging data of the aging characteristic indices: (**a**) $P^{1}$; and (**b**) $P^{2}$.

**Figure 12:**
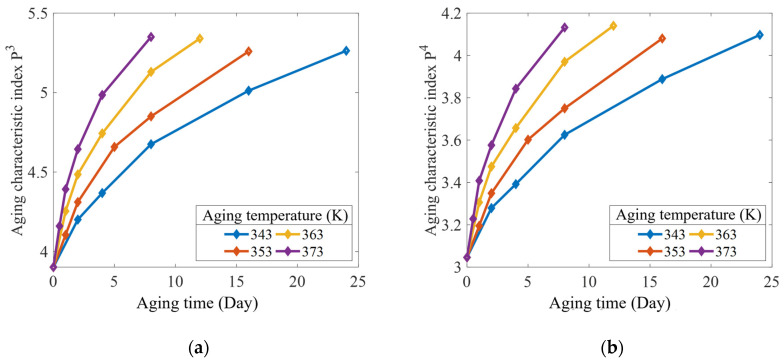
Accelerated aging data of the aging characteristic indices: (**a**) $P^{3}$; and (**b**) $P^{4}$.

According to the steps of the prediction model for the crack propagation rate interval of the aging rubber, which were established in Section 3.2.2, the parameters ($-\ln a_{1},-\ln a_{2},b_{1},b_{2}$) were used as the aging characteristic indices $P^{j}$. The relationships between the aging characteristic index, accelerated aging temperature, and aging time are shown in Figure 11 and Figure 12.

Under the action of each accelerated temperature stress, a linear regression analysis was performed on the decay trajectory of each aging characteristic index with aging time using Equation (22). The coefficients of the decay trajectory function were obtained through fitting curves, as listed in Table 5. To ensure the consistency of the rubber material’s degradation mechanism under the accelerated test temperature, according to the data in Table 5, the activation energy at each acceleration temperature was determined using Equation (24), as listed in Table 6. The results show that the maximum deviation between the activation energy and the mean value was 7.4% in the range of the acceleration test temperature. The effectiveness of the accelerated test was illustrated by applying the Arrhenius equation (Equation (23)). Table 7 shows the temperature acceleration model coefficients obtained for each aging characteristic index. Notably, each correlation coefficient was greater than 0.95, thereby indicating a good fitting effect.

Thus, the evolution of the aging characteristic indices with aging time (*t*) at different temperatures (*T*) was obtained as follows:

(27) $$\ln a_{1}=-P^{1}=-\exp(3.689+\exp(4.913-2891.3/T)\cdot t^{0.575})$$

(28) $$\ln a_{2}=-P^{2}=-\exp(3.516+\exp(5.078-3091.9/T)\cdot t^{0.650})$$

(29) $$b_{1}=P^{3}=\exp(1.358+\exp(4.747-2577.6/T)\cdot t^{0.500})$$

(30) $$b_{2}=P^{1}=\exp(1.111+\exp(4.678-2579.1/T)\cdot t^{0.518})$$

### 4.3. Verification and Application

This study verified the accuracy of the interval prediction model for rubber crack propagation, considering the effects of aging, by conducting a fatigue cyclic tensile test on rubber materials aged at 333 K for 20 d. The validity of the proposed prediction model was determined by comparing the description area of the interval parameter correlation model’s (IPCM) prediction results with that of the crack propagation rate test results for the aging rubber and the description area of the independent interval variable model’s (IIVM) prediction results.

The rubber performance parameters under the verification test conditions were predicted using the prediction model of the rubber crack propagation parameter limits obtained in Section 4.2 (Equations (27)–(30)). The predicted results for each parameter were obtained as follows:

(31) $$\ln a_{1}=-44.605,\ln a_{2}=-36.100,b_{1}=4.926,b_{2}=3.784$$

(32) lna∈[−44.605,−36.100],b∈[3.784,4.926]

Using Equation (19), the boundary equation parameters of the aging rubber were obtained as follows:

(33) $$\{\begin{array}{c} b_{3}=\frac{Y_{1}(\ln G_{m})-Y_{2}(\ln G_{1})}{\ln G_{m}-\ln G_{1}}=4.877\\ \ln a_{3}=\frac{\ln G_{m}\cdot Y_{1}(\ln G_{1})-\ln G_{1}\cdot Y_{2}(\ln G_{m})}{\ln G_{m}-\ln G_{1}}=-41.897\\ b_{4}=\frac{Y_{2}(\ln G_{m})-Y_{1}(\ln G_{1})}{\ln G_{m}-\ln G_{1}}=4.043\\ \ln a_{4}=\frac{\ln G_{m}\cdot Y_{2}(\ln G_{1})-\ln G_{1}\cdot Y_{1}(\ln G_{m})}{\ln G_{m}-\ln G_{1}}=-39.480 \end{array}$$

The parameter values in Equations (31) and (33) were introduced into the interval parameter correlation model of the rubber crack propagation (Equation (20)). The variation range relation of the crack propagation parameters after the rubber aging in the verification group was obtained using the following equation:

(34) $$\{\begin{array}{c} b\in [3.784,4.043],\ln a=[-15.093-b\cdot 5.991,-1.355-b\cdot 8.780]\\ b\in [4.043,4.877],\ln a=[-15.093-b\cdot 5.991,-13.430-b\cdot 5.991]\\ b\in [4.877,4.926],\ln a=[-2.876-b\cdot 8.780,-13.430-b\cdot 5.991] \end{array}$$

**Figure 13:**
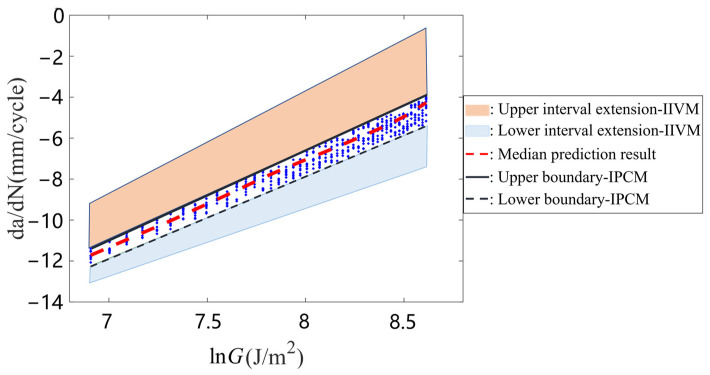
Comparison of trial results with predicted results..

Finally, the crack propagation areas of the aging rubber in the validation group were described using the IPCM and IIVM, respectively. The results of these models were compared to those of the fatigue test on the aging rubber in the verification group, as shown in Figure 13 (IPCM was used to establish an interval parameter correlation model, i.e., Equation (34); IIVM considered the crack propagation parameters as independent interval variables, i.e., Equation (32)).

As can be seen from the comparison results shown in Figure 13, the interval obtained by IPCM effectively covered the rubber fatigue test results of the verification test. Within the description range of the rubber crack propagation interval parameters, the maximum error between the prediction and test results on the upper boundary of the rubber crack propagation rate was 14%, and that on the lower boundary of the rubber crack propagation rate was 18%. However, the maximum error between the predicted results of the rubber crack propagation parameters based on the average value and experimental results was 80%. The boundary error of the prediction results based on the interval parameters was considerably smaller than that of the average prediction results.

## 5. Conclusions

This study developed a method for predicting the variational range of the crack propagation performances of the aging CR materials of an air spring airbag. In the prediction method, an interval prediction model, considering material aging for a determination of the crack propagation parameters of the CR, was proposed. This provided a means of application for fatigue crack propagation life reliability research on air spring airbags with a long service life. The test results showed that, with the aging of the rubber, the rubber material hardened and its anti-fatigue property decreased greatly. The validity of the predictive model was verified by comparing the rubber fatigue crack propagation test results obtained via the validation test with the region described by the rubber crack propagation parameter interval prediction results, and the following conclusions were obtained.

(1) A comparison of the test results with the prediction results shows that the maximum deviation between the predicted and experimental values for the median crack propagation rate of the aged rubber was greater than or equal to 80%. However, when the crack propagation rate interval was considered in the characteristic equation, the maximum deviation between the experimental and calculated values was significantly reduced to less than 18%. Therefore, the deterioration behavior of the aging rubber’s fatigue crack propagation rate could be predicted with more accuracy using the rubber crack propagation rate interval prediction equation.
(2) By applying the parameter correlation equation, the rubber’s fatigue crack propagation characteristics were described more accurately. Compared to the independent interval parameter analysis method, the proposed method effectively avoided the interval expansion problem caused by independent interval parameters. Moreover, it eliminated the inconsistency between the parameter variation range caused by the interval parameter optimization method and the actual situation.

The prediction model in this study was established based on test data at constant temperature (296 K), only considering the influence of this temperature on the rubber aging. Therefore, the premise of the application of this model is to meet the requirement that a change in ambient temperature will not directly affect the properties of rubber materials. It is worth noting that this paper only demonstrated the effectiveness of the proposed prediction model in predicting the fatigue crack propagation characteristic interval of air spring bag rubber (CR) after aging. By a suitable extension, the method may be applied to the prediction of other kinds of rubber products under uncertainties and air aging.

## Figures and Tables

**Table 1:** Main mechanical properties of unaged rubber.

Mechanical Properties	Values
Shore hardness (Ha)	56
Tensile strength (MPa)	16.8
Ductility (%)	640

**Table 2:** Material composition.

Components	Proportion (phr)
CR	100
Carbon black	30
Zinc oxide	5
Magnesium oxide	4
Stearic acid	1
Accelerator	2.5
Diphenylamine antioxidant	3
P-phenylenediamine antioxidant	3

**Table 3:** Aging parameters.

Aging Temperature (K)	Durations of Aging (Days)
343	0, 2, 4, 8, 16, 24
353	0, 1, 2, 5, 8, 16
363	0, 1, 2, 4, 8, 12
373	0, 0.5, 1, 2, 4, 8

**Table 4:** Crack propagation parameter values of accelerated aging rubber.

Aging Temperature (K)	Duration of Aging (Days)	Crack Propagation Parameter			
		$\ln a_{1}$	$\ln a_{2}$	$b_{1}$	$b_{2}$
343	0	−40.101	−33.697	3.902	3.046
	2	−41.550	−34.656	4.201	3.279
	4	−42.432	−35.122	4.368	3.392
	8	−44.338	−36.469	4.675	3.625
	16	−46.368	−37.929	5.012	3.888
	24	−48.003	−39.251	5.263	4.097
353	1	−40.933	−34.113	4.105	3.195
	2	−42.085	−34.875	4.311	3.349
	5	−44.146	−36.237	4.658	3.602
	8	−45.295	−37.041	4.850	3.750
	16	−47.884	−39.030	5.259	4.080
363	1	−41.844	−34.750	4.254	3.306
	2	−43.076	−35.511	4.485	3.475
	4	−44.623	−36.476	4.743	3.657
	8	−47.043	−38.333	5.130	3.970
	12	−48.359	−39.341	5.340	4.140
373	0.5	−41.223	−34.238	4.160	3.228
	1	−42.479	−35.111	4.392	3.408
	2	−43.967	−35.965	4.644	3.576
	4	−46.089	−37.520	4.985	3.843
	8	−48.371	−39.213	5.350	4.132

**Table 5:** Fitting results of dynamic curve parameters for each accelerated aging characteristic index.

Aging Characteristic Index	$\ln P_{0}$	K				α
		343	353	363	373	
$P^{1}$	3.689	0.0299	0.0378	0.0462	0.0594	0.575
$P^{2}$	3.516	0.0198	0.0249	0.0315	0.0410	0.650
$P^{3}$	1.358	0.0630	0.0780	0.0932	0.1162	0.500
$P^{4}$	1.111	0.0585	0.0723	0.0869	0.1079	0.518

**Table 6:** Calculation results of activation energy for each accelerated temperature interval.

	Temperature Range			
	343 to 353	353 to 363	363 to 373	Average Value
$E_{a}$ of $P^{1}$	23,628	21,463	24,435	23,175.3
$E_{a}$ of $P^{2}$	23,009	25,217	23,931	24,052.3
$E_{a}$ of $P^{3}$	21,514	19,921	21,932	21,122.3
$E_{a}$ of $P^{4}$	21,253	20,551	21,377	21,060.3

**Table 7:** Fitting results of Arrhenius equation parameters for each aging feature.

	lnZ	$E_{a}/R$	Correlation Coefficient
$P^{1}$	4.913	2891.3	0.956
$P^{2}$	5.078	3091.9	0.952
$P^{3}$	4.747	2577.6	0.960
$P^{4}$	4.678	2579.1	0.972

## Data Availability

The data presented in this study are available on request from the corresponding author. The details of the proposed methodology and of the specific values of the parameters considered have been provided in the paper. Hence, we are confident that the results can be reproduced.

## References

[1] Mars W., Fatemi A. (2002). A literature survey on fatigue analysis approaches for rubber. Int. J. Fatigue.

[2] Tee Y.L., Loo M.S., Andriyana A. (2018). Recent advances on fatigue of rubber after the literature survey by Mars and Fatemi in 2002 and 2004. Int. J. Fatigue.

[3] Moon B., Jun N., Park S., Seok C.-S., Hong U.S. (2019). A Study on the Modified Arrhenius Equation Using the Oxygen Permeation Block Model of Crosslink Structure. Polymers.

[4] Wang Z. (2021). Research on fatigue failure mode and failure theory of rubber. J. Phys. Conf. Ser..

[5] Young D.G. (1990). Application of fatigue methods based on fracture mechanics for tire compound development. Rubber Chem. Technol..

[6] Qiu X., Yin H., Xing Q. (2022). Research Progress on Fatigue Life of Rubber Materials. Polymers.

[7] Gent A.N., Lindley P.B., Thomas A.G. (1964). Cut propagation and fatigue of rubber. I. The relationship between cut propagation and fatigue. J. Appl. Polym. Sci..

[8] De D.K., Gent A.N. (1998). Crack growth in twisted rubber disks. Part II: Experimental results. Rubber Chem. Technol..

[9] Rivlin R.S., Thomas A.G. (1953). Rupture of rubber. I. Characteristic energy for tearing. J. Polym. Sci..

[10] Peng Y., Liu G., Quan Y., Zeng Q. (2016). Cracking energy density calculation of hyperelastic constitutive model and its application in rubber fatigue life estimations. J. Appl. Polym. Sci..

[11] Wu J., Chen L., Chen D., Wang Y., Su B., Cui Z. (2021). Experiment and Simulation Research on the Fatigue Wear of Aircraft Tire Tread Rubber. Polymers.

[12] Senthilvel K., Rathinam N., Prabu B., Kumar A.A.J. (2020). Investigation on the mechanical and ageing properties of acrylic rubber reinforced halloysite nanotubes/carbon black hybrid composites. J. Elastomers Plast..

[13] Porter C., Zaman B., Pazur R. (2022). A critical examination of the shelf life of nitrile rubber O-Rings used in aerospace sealing applications. Polym. Degrad. Stab..

[14] Tang N., Soltani P., Pinna C., Wagg D., Whear R. (2018). Ageing of a polymeric engine mount investigated using digital image correlation. Polym. Test..

[15] Kang W., Liu J., Xiong W., You T., Wang X., Zeng K., Deng Y., Guo Z., Yuan K. (2022). Basic mechanical and fatigue properties of rubber materials and components for railway vehicles: A literature survey. Rev. Adv. Mater. Sci..

[16] Liu Q., Shi W., Chen Z., Li K., Liu H., Li S. (2019). Rubber accelerated ageing life prediction by Peck model considering initial hardness influence. Polym. Test..

[17] Han S.W., Kwak S.B., Choi N.S. (2014). Accelerated life prediction of ethylene-propylene diene monomer rubber subjected to combined degradation. Trans. Korean Soc. Mech. Eng. A.

[18] Kim W.S., Woo C.S., Cho S.J., Kim W.D. (2002). Prediction of useful life by heat aging of motor fan isolating rubber. Elastomers Compos..

[19] Olejnik A., Smejda-Krzewicka A., Strzelec K. (2019). Effect of antioxidants on aging of the chloroprene rubber/butadiene rubber (CR/BR) blends. Int. J. Polym. Anal. Charact..

[20] Kashi S., Varley R., De Souza M., Al-Assafi S., Di Pietro A., de Lavigne C., Fox B. (2018). Mechanical, Thermal, and Morphological Behavior of Silicone Rubber during Accelerated Aging. Polym.-Plast. Technol. Eng..

[21] Moon B., Kim K., Park K., Park S., Seok C.-S. (2020). Fatigue life prediction of tire sidewall using modified Arrhenius equation. Mech. Mater..

[22] Moon B., Lee J., Kim S., Park S., Seok C.-S. (2022). Methodology for Predicting the Durability of Aged Tire Sidewall Under Actual Driving Conditions. Int. J. Precis. Eng. Manuf..

[23] Chen L., Rao S. (1997). Fuzzy finite-element approach for the vibration analysis of imprecisely-defined systems. Finite Elem. Anal. Des..

[24] Elishakoff I. (1998). Three Versions of the Finite Element Method Based on Concepts of Either Stochasticity, Fuzziness, or Anti-Optimization. Appl. Mech. Rev..

[25] Wu J., Zhang Y., Chen L., Luo Z. (2013). A Chebyshev interval method for nonlinear dynamic systems under uncertainty. Appl. Math. Model..

[26] (2011). Rubber, Vulcanized or Thermoplastic—Accelerated Ageing and Heat Resistance Tests.

[27] Gillen K.T., Bernstein R., Derzon D.K. (2005). Evidence of non-Arrhenius behaviour from laboratory aging and 24-year field aging of polychloroprene rubber materials. Polym. Degrad. Stab..

[28] Lee J.H., Bae J.W., Kim J.S., Hwang T.J., Park S.D., Park S.H., Yeo T.M., Kim W., Jo N.J. (2011). Life-time prediction of a chloroprene rubber (CR) O-ring using intermittent compression stress relaxation (CSR) and time-temperature superposition (TTS) principle. Macromol. Res..

[29] (2015). Standard Test Methods for Vulcanized Rubber and Thermoplastic Elastomers.

[30] Mullins L. (1969). Softening of Rubber by Deformation. Rubber Chem. Technol..

[31] Yeoh O.H. (2001). Analysis of deformation and fracture of pure shear rubber test piece. Plast. Rubber Compos..

[32] Béranger A.S., Qin J., Heuillet P., Baurier H. (2018). Fatigue crack propagation behavior of NBR, HNBR, HNBR ZSC compounds. Procedia Eng..

[33] (2014). Standard Test Method for Rubber Deterioration Crack Growth.

[34] Lake G.J. (1995). Fatigue and Fracture of Elastomers. Rubber Chem. Technol..

[35] Liu Q., Shi W., Li K., Chen Z., Liu H. (2019). Performance degradation prediction and reliability evaluation of rubber aging in natural environment under alternating cyclic thermal load. IEEE Access.

[36] Arrhenius S. (1967). On the reaction velocity of the inversion of cane sugar by acids. Sel. Read. Chem. Kinet..

